# Cysticercus in the Human Brain

**Published:** 1853-08

**Authors:** A. Brunniche


*Cysticercus in the Human Brain.* By A. BRUNNICHE.—A servant girl, aged 43, of healthy frame and aspect, but for three years subject to epileptic fits, came to the hospital May 3, 1851, suffering from supposed ague. Upon admission, her symptoms were, localised pain all over the left part of the forehead and temples, weakness in the muscles of the face, sparks and flashes of light in the right eye, occasional dilatation of the pupil, squinting, marked diminution of the intellectual capacity, combined with erroneous ideas and hallucinations, slow, unconnected utterance, frequent vomiting, involuntary discharge of fæces, slow pulse, and weakness in the lower extremities. She had often complained of cold, and want of circulation in the fingers; so that, at the commencement, her illness had been mistaken for ague. She died May 21.

*Examination of the Body.*—Skull-cap thinner than natural; the surface of the brain bulged forwards from the cranial cavity; the membranes were thin, but full of blood. There were three cysticerci within the cranium. The largest lay in the substance of the anterior lobe of the left hemisphere in a cavity the size of a hen's egg, projecting into the anterior part of the lateral ventricle, and making a marked depression into the corresponding corpus striatum. One worm was contained in this cavity, but in no way connected with the wall. It was surrounded by a sac as large as a hazel-nut, whence proceeded other similar, but distinct prolongations. A second worm was found in the middle lobe of the left hemisphere, near the preceding; and a third in the posterior part of the right hemisphere, both invested in a surrounding membrane. The three worms were unconnected with one another, and in no organic union with surrounding parts; yet they had formed for themselves a well-organised capsule or bag. As regards the question, how these animals or their ova found their way into the human organism, the author believes that they must have been conveyed to the brain by the current of blood, after being taken up from the free surface of the body. Some authors have, in these cases, remarked no cerebral symptoms. In this there was marked disturbance of the intellectual faculties, although the substance of the brain was not in any way altered.—*Med. Times and Gaz.* from *Hospitals Meddelelser.*

